# Evening smartphone exposure impairs sleep quality and next-day performance in elite soccer players: a randomized controlled trial

**DOI:** 10.5114/biolsport.2026.152348

**Published:** 2025-08-29

**Authors:** Nadia Dridi, Mohamed Abdelkader Souissi, Rim Dridi, Halil İbrahim Ceylan, Nicola Luigi Bragazzi, Atef Salem, Sofien Fekih, Mokhtar Chtara, Bessem Mkaouer, Hamdi Chtourou, Ismail Dergaa, Nizar Souissi, Valentina Stefanica, Piotr Żmijewski, Ryland Morgans

**Affiliations:** 1 Physical Activity, Sport and Health Research Unit (UR18JS01), National Observatory of Sports, Tunis 1003, Tunisia; 2 High Institute of Sport and Physical Education, Ksar Said, University of Manouba, Mannouba 2010, Tunisia; 3 High Institute of Sport and Physical Education of Gafsa, Gafsa University, Gafsa, Tunisia; 4 Research Laboratory (LR23JS01) “Sports Performance, Health, and Society, Mannouba 2010, Tunisia; 5 Physical Education and Sports Teaching Department, Faculty of Sports Sciences, Ataturk University, Erzurum, Turkey; 6 Laboratory for Industrial and Applied Mathematics (LIAM), Department of Mathematics and Statistics, York University, Toronto, ON M3J 1P3, Canada; 7 Scientific Center of Research and Sports Performance, Sharjah Women’s Sports, United Arab Emirates; 8 High Institute of Sport and Physical Education Sfax, University of Sfax, Sfax 3000, Tunisia; 9 Department of Physical Education and Sport, Faculty of Sciences, Physical Education and Informatics, National University of Science and Technology Politehnica Bucharest, Pitesti University Center, Pitesti, Romania; 10 Institute of Sport-National Research Institute, Warsaw, Poland; 11 School of Sport and Health Sciences, Cardiff Metropolitan University, Cardiff, UK

**Keywords:** Athletic performance, Blue light, Circadian rhythm, Melatonin, Psychometrics, Psychomotor performance, Recovery, Screen time, Sleep hygiene

## Abstract

This study aimed to examine the effects of pre-bedtime smartphone use on sleep quality and athletic performance in soccer players while also investigating potential time-of-day variations. In this randomized controlled crossover trial, 16 male elite-level players were assigned to either use a smartphone for two hours prior to bedtime or read magazines (control), separated by a one-week washout period. Participants completed morning and afternoon performance tests (cognitive and physical assessments) and sleep quality measurements. Nocturnal smartphone use significantly impaired sleep quality, increasing sleepiness after days 3 and 5 (p < 0.01; d=5.74, d=5.72, respectively), decreasing total sleep time, increasing sleep onset latency, and reducing sleep efficiency (all p < 0.01; d=1, d=4.59). Cognitive performance initially showed improved afternoon results, although following five days of smartphone use, this pattern reversed with enhanced morning performance (p < 0.01; d=0.53, d=1.48). Simple and choice reaction times deteriorated significantly in afternoon sessions compared to both baseline and control conditions (p < 0.01; d=0.96–3.47). Physical performance tests revealed decreased jumping ability and slower reactive agility times following five nights of smartphone use, particularly in afternoon sessions (p < 0.01; d=0.85–0.91). Five consecutive nights of pre-bedtime smartphone use impaired sleep quality and both cognitive and physical performance in elite soccer players, with stronger effects in afternoon sessions. These findings emphasize the importance of implementing device-free periods prior to bedtime and potentially adjusting training schedules when evening screen exposure is unavoidable. Future research should explore countermeasures for managing evening device exposure in elite athletes.

## INTRODUCTION

Sleep is a crucial component of athletic recovery as well as physical and psychological performance. Previous studies demonstrated strong positive associations between sleep and various aspects of athletic performance, including sports-specific skills, strength, and muscular power [1, 2]. These benefits were further supported by Mah et al. [1], who found that adequate sleep improved sprint times, reaction times, accuracy, and mood state.

The athlete’s sleep is influenced by both sport-specific factors – such as training schedules, travel, and competition demands – and non-sport factors, including stress and anxiety [2]. The available body of evidence indicates that elite athletes are particularly susceptible to sleep inadequacies, which are characterized by habitual short sleep duration (< 7 hours each night) and poor sleep quality, for instance, fragmented sleep [2]. Although insufficient sleep can significantly impair athletic performance through multiple mechanisms, individual differences in susceptibility to sleep disturbances may modulate the extent of these effects among soccer players. At the physiological level, sleep deprivation negatively affects muscle glycogen stores and increases perceived stress [3, 4], while also reducing time to exhaustion [5] and decreasing movement accuracy [6, 7]. Beyond physical effects, inadequate sleep impairs cognitive function, emotional regulation, and decision-making abilities [8–11], while also contributing to mood disturbances [12]. Moreover, chronobiological factors such as individual chronotype and circadian phase alignment play a crucial role in athletic performance and recovery [13]. Evidence shows that evening chronotypes tend to experience greater sleep deficits and impaired morning performance, which is particularly relevant for athletes with early training schedules [14, 15].

Although athletes and coaches believe that adequate sleep is essential for optimal performance [16–18], there are many situations when sleep disruptions occur prior to major competitions, such as early morning training, increases in training load, travel departure times and performing at altitude [19]. Athletes who reported worsened sleep (quality and/or quantity) prior to competition suggested nervousness and negative thoughts regarding the competition contributed to sleep problems [20]. Furthermore, one of the major contributing external factors in sleep decline is the use of light-emitting devices (such as tablets, televisions, smartphones, computers) specifically during the hours prior to sleep [21], as light is considered the most potent environmental signal that impacts the human circadian clock [22]. Additionally, blue-light exposure may also lead to a phase shift in the biological clock and acutely suppresses the sleepfacilitating hormone melatonin [21, 23, 24], thus leading to a reduction in overall sleep quality and quantity [25–28]. However, other potential factors that may affect sleep, such as stress levels [29], dietary habits, or stimulant intake [30], should also be considered when interpreting the observed effects.

The use of electronic devices in the evening has been associated with more perceived difficulty in falling asleep [26, 31]. It has been shown that exposure to the blue light from smartphone LED displays during night-time may impair sleep quality and increase errors of commission. These effects were observed through a delayed onset of melatonin and a rise in body temperature, although these physiological changes did not reach statistical significance [32]. However, the findings did suggest that alterations in sleep and cognitive functions may serve as more sensitive indicators of blue light exposure from smartphones than changes in melatonin, cortisol, or body temperature [32]. Other studies showed no effect of short-wavelength light from smartphones and melatonin tablets release [28, 33]. Further studies have shown athletes commonly engage in blue lightemitting activities prior to bedtime [34]. Jones et al. [35] found that the number of electronic devices used prior to bed was associated with athletes’ perceived sleep onset time, although no relationship between device use and actual bedtime or time spent in bed was observed. Interestingly, Jones et al. [36] demonstrated that removing electronic devices in the evening during short-duration training camps (4–7 nights) did not lead to improvements in either sleep quantity or cognitive performance. Considering the psychological effects of cell phone usage, it has been reported that a correlation between smartphone addiction and sleep disturbance has been found [37], which can potentially be a consequence of an increase in sleep disorders and fatigue. A further study showed that participants with poor sleep quality had a greater degree of smartphone dependence than participants with good sleep quality. In particular, lower smartphone dependence and enhanced health-related behaviors, resulted in improved sleep quality [38]. Emerging research indicates that elite youth athletes increasingly engage in evening screenbased activities, particularly via smartphones and tablets, often as part of recovery routines or social interactions [39, 40]. However, such habits may inadvertently contribute to sleep disturbances due to overstimulation and circadian disruption.

However, to the best of the authors’ knowledge, there are limited studies investigating the combined effects of evening electronic device use on sleep quality, cognitive performance, and physical performance in soccer players. Additionally, the time-of-day effects of such exposure on athletic performance remain largely unexplored. Furthermore, while the acute effects have been studied, the cumulative impact of consecutive nights of device use on athletic performance has not been thoroughly examined. Thus, based on these gaps in the existing literature regarding the cumulative effects of evening device use and the lack of comprehensive studies examining both cognitive and physical performance measures in athletes, the study aims are to: (i) examine how smartphone usage two hours prior to bed for five consecutive nights affects young soccer players’ sleep quality, (ii) assess its impact on both cognitive and physical performance, and (iii) investigate potential time-of-day variations in these effects. Based on existing literature [43, 44], the study hypothesis is that using a smartphone two hours prior to bedtime will impair the sleep quality of elite soccer players, resulting in increased sleep latency and reduced total sleep duration. Furthermore, it is predicted that this sleep disruption will lead to declines in cognitive performance, such as attention and reaction time, as well as physical performance, including reactive agility and explosive strength. Finally, these effects will be more pronounced in the morning, when participants are still affected by cumulative sleep deprivation.

## MATERIALS AND METHODS

### Study design

Monday, Wednesday, and Friday preceding the experiment, participants completed three familiarity sessions considering procedures, experimenters, and tests. The experimental protocol consisted of two randomized trials separated by one week. For each trial, participants engaged in a specific activity (either watching videos or reading magazines) for two hours before bedtime (between 8:00 PM and 10:00 PM) across five consecutive nights. A researcher was present during each session to monitor adherence to the protocol and ensure standardization. The researchers responsible for administering the cognitive and physical performance tests were blinded to the participants’ assigned condition (video or magazine) in order to minimize assessment bias.

In the experimental condition, participants freely used an 11-inch Lenovo Yoga Tab 11 tablet between 8:00 PM and 10:00 PM. The video content was standardized across all conditions to ensure consistency and minimize variability. It was selected for its neutral entertainment value, avoiding any elements that could engage participants cognitively or emotionally. The tablet’s blue light was quantified using a spectroradiometer (Model PR-655, Photo Research, Chatsworth, CA, USA). The measured blue light had a peak wavelength of 460 nm and an intensity of 30 μW · cm−2 when viewed from a distance of 50 cm [26]. During each blue light exposure session, the same video was presented on the tablet for viewing. To prevent any further exposure, the tablet, along with all other electronic devices, was immediately removed at the end of each session. The videos were selected based on neutral entertainment value, defined as content devoid of emotionally or cognitively stimulating material (e.g., no dramatic events, moral dilemmas, or complex narratives). To ensure standardization, all videos were matched in duration, format, and thematic neutrality (e.g., nature scenes, slow-paced documentaries, or basic lifestyle segments).

In the control condition, participants read printed magazines, selected for non-engaging and neutral entertainment nature, between 8:00 PM and 10:00 PM, to minimize cognitive and emotional stimulation [25]. If participants chose not to read, instructions were provided to relax while avoiding falling asleep for two hours. This activity was designed to serve as a standardized control condition with minimal cognitive engagement. Efforts were made to control for cognitive load by selecting neutral content, ensuring that the primary difference between the conditions stemmed from the type of media exposure rather than the content itself.

Both conditions were conducted in a controlled environment with standardized light intensity, room temperature, and noise levels. Lighting was controlled by conducting all experimental sessions in a standardized environment with artificial lighting. To ensure consistency, participants were required to maintain regular sleeping schedules, which were recorded in a sleep diary, and to follow dietary habits. Participants consumed a standardized dinner 3.5 hours prior to habitual bedtime and refrained from consuming caffeine, alcohol, or other substances that might influence alertness or sleep quality. Participants were also instructed to maintain habitual physical activity levels while avoiding strenuous exercise in the 24 hours preceding testing sessions. Training loads were standardized and monitored throughout the study period using rate of perceived exertion (RPE) scores and duration of training sessions, with all participants following the same team training schedule to ensure consistency in physical exertion levels between experimental conditions.

**FIG. 1:**
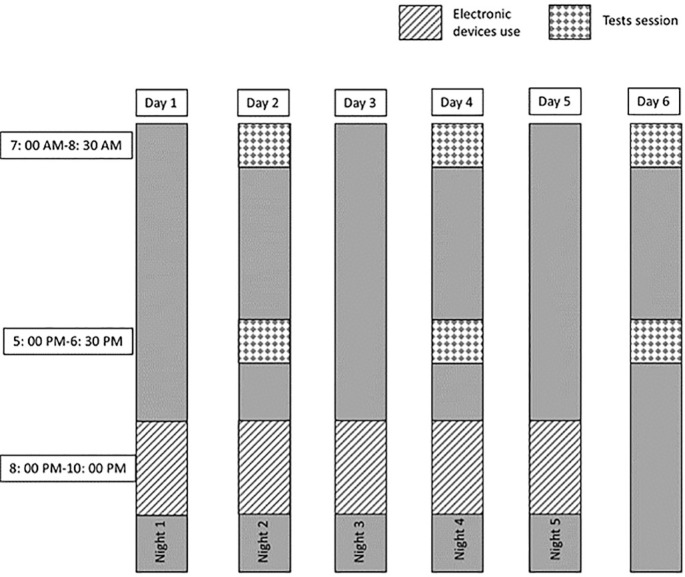
Experimental design of the study.

After the first, third, and fifth nights in both conditions, participants underwent identical testing sessions conducted in the morning (between 7: 00 AM and 8: 30 AM) and afternoon (between 5: 00 PM and 6: 30 PM) (Figure 1). These timings were selected based on literature documenting the phases of minimum and maximum daytime levels of short-term maximal performance [41–43]. The testing sessions included (i) cognitive performance tasks such as Simple Reaction Time (SRT), Choice Reaction Time (CRT), Trail Making Test (TMT), and The Digit Cancellation Test (D-CAT), (ii) physical performance tests, including squat jump (SJ), countermovement jumps with arm swing (CMJA), and Reactive Agility Test (RAT). Recovery periods were standardized to five minutes between physical tests and two minutes between trials of the same test.

To minimize potential confounding factors, participants adhered to strict dietary guidelines. Prior to morning testing, participants were limited to one glass (150–200 mL) of water to avoid postprandial thermogenesis effects. Prior to afternoon testing, participants consumed an individualized isocaloric meal at 12: 00 midday, standardized in macronutrient composition and representing approximately one-third of the participants’ estimated daily energy requirements. These requirements were calculated using the Mifflin-St Jeor equation based on each participant’s age, weight, height, and physical activity level. Following the meal, water consumption was permitted ad libitum. Compliance with these protocols was easily ensured, as all participants were members of the same soccer team.

### Participants

Sixteen male soccer players competing at a high-performance level (mean (SD) age 19.75 ± 1.0 y, range 18–21 y; height 1.75 ± 0.05 m; body mass 66.75 ± 3.19 kg) volunteered to participate in this study. All participants were adults (≥ 18 years) at the time of enrollment, meeting ethical requirements for informed consent without parental approval.

The players were affiliated with a professional club competing in the Tunisian Premier League, with players performing five training sessions per week. Participants had more than eight years of experience in soccer, a criterion commonly used to classify players as high-level athletes [44]. Following a thorough explanation of the purpose, methods, and possible risks and benefits of the study, participants provided written informed consent prior to participation. All participants had regular sleep schedules (sleep duration 7.5 ± 0.5 hours) based on the Bastuji and Jouvet [45] calendar, completed over one month. To have a group without extreme morning or evening types, participants were selected as “neither type” based on answers to the Horne and Östberg self-assessment questionnaire [46]. During the experiment period, no lower-limb injuries or muscle soreness were observed and no participant was consuming medication.

Players were classified as centre-backs (CB; n = 10), full-backs (FB; n = 11), centre midfielders (CM; n = 8), wingers (W; n = 5), and centre forwards (CF; n = 7). If a player fulfilled multiple playing positions during match-play, the player was categorized accordingly to each position [13]. Although playing positions were recorded, no subgroup analyzes based on this variable were conducted due to the limited sample size and the study’s focus on the overall effects of the intervention across all players. The required sample size was calculated using G*Power software (version 3.1.9.6; Kiel University, Kiel, Germany) to ensure a statistical power (1−β) of 0.80 and a significance level (α) of 0.05. A large effect size (Cohen’s d = 0.85) was selected, in line with findings from Dergaa et al. [47], who reported a comparable effect size (d = 0.85) for improvements in perceived sleep quality following a recovery intervention in physically active young males. Furthermore, the effect sizes observed in our own data for key outcomes such as jump height and reactive agility after smartphone use were within a similar range (d = 0.85–0.91), supporting the validity of this estimate. Based on these parameters, the power analysis determined that a minimum of 14 participants was required.

### Ethical Approval

The study protocol adhered to the ethical principles outlined in the Declaration of Helsinki and was approved by the Ethics Committee of the Faculty of Medicine of Sfax, University of Sfax, Sfax, Tunisia (reference number 49/2024). The study also complied with the ethical and procedural requirements of the conduct of sports medicine and exercise science research outlined by Guelmemi et al. [48].

### Testing procedures

#### Cognitive Performance Tests

##### Simple Reaction Time (SRT)

The simple reaction time (SRT) involved the appearance of a green circle on a black background, positioned horizontally at the center of the computer screen. Participants were instructed to press a button with the preferred thumb as quickly as the imperative stimulus appeared on the screen. The imperative visual signal was preceded by a preparatory warning visual signal (a white circle) that appeared immediately prior to the imperative signal. The duration of the preparatory period was variable and random, ranging between 200 and 1400 milliseconds (ms) and commenced with the participant’s previous response.

The SRT task was performed at rest and was presented 20 times during each experimental session.

### Choice Reaction Time (CRT)

In this type of task, the preparatory visual signal only served as a warning and did not provide information regarding the location of the imperative signal. The response CRT task involved pressing a button with either the left or right thumb, depending on the spatial configuration of the imperative signal. For example, if the signal appeared on the upper right, the participant had to press the right button located at the top of the screen. Thus, the participant faced a processing alternative and had to continuously select a response choice.

The imperative signal was preceded by a preparatory visual signal consisting of four white circles arranged in a square pattern, with a randomized onset between 200 and 1400 ms. Participants were instructed to press the appropriate button as quickly as possible when one of the four circles turned green. Reaction time was recorded for each trial with higher scores reflecting slower performance. An error was defined as either an incorrect response (i.e., pressing the wrong button) or an anticipatory response. Both reaction time tests were conducted using the free software OpenSesame [49]. In fact, meta-analyzes of standard CRT tasks report generally low reliability, with Pearson correlations indicating suboptimal stability across sessions (0.30–0.37) [50].

### Trail Making Test (TMT)

Two tests were conducted to assess visual attentional control, perceptual speed, and working memory capacity. Both tests were performed in a seated position. The first component of the trail-making test (TMT-A) evaluates visuo-perceptual abilities, while the TMT-B assesses working memory and task-switching ability [51, 52]. Both parts were done with a pencil and paper. In part A, numbers from 1 to 25 have to be linked using a pencil; for part 2, the number sequence is interrupted each time by the corresponding letter (1–13; A–L) [52]. Completion time (in seconds) was recorded to the nearest 0.1 s using a digital stopwatch [53]. The TMT B-A score (which was calculated as the difference between the TMT-B and the TMT-A completion times) is thought to provide a relatively pure indicator of cognitive flexibility [52], which is a fundamental component of executive control and refers to one’s ability to efficiently switch between tasks [54].

Participants performed the TMT under two experimental conditions: (1) evening electronic device use and (2) a control condition. To control for potential learning or fatigue effects, the order of test administration was systematically counter-balanced. Specifically, participants were randomly assigned to one of two test orders: half completed TMT-A first, followed by TMT-B, while the other half completed TMT-B first, followed by TMT-A. This approach ensured that any order effects were evenly distributed across conditions.

Moreover, the TMT has demonstrated good test-retest reliability in previous studies [55–57], with reported reliability coefficients typically ranging from 0.71–0.89 for TMT-A, and 0.74–0.94 for TMT-B, and approximately 0.74 for the B-A difference score. These values indicate moderate to high stability across repeated administrations, supporting the use of the TMT in repeated-measures experimental designs [55].

### Attention Task (Digit cancellation test)

Attention was assessed using a pencil-and-paper test validated by Zazzo [58]. This is a visual discrimination task (detection of target numbers every time it finds one within a series of lines of numbers) [59]. The Digit Cancellation Test (D-CAT) demonstrates high test-retest reliability, with correlation coefficients of 0.79 for the single-target trial, 0.86 for the two-target trial, and 0.85 for the three-target trial, thus all statistically significant and indicating strong stability across sessions [60].

### Physical Performance Tests

#### Vertical Jump tests

Participants performed maximal vertical jumps (SJ) and countermovement jumps with arm swing (CMJA) using an infrared jump system (Optojump, Microgate, Bolzano, Italy) connected to a microcomputer. The Optojump system measures flight and contact times with an accuracy of 1/1000 of a second. The SJ consisted of a maximal jump from a flexed knee position (approximately 90°), with hands on the hips, without performing any countermovement prior to the start of the jump. The CMJA involved a leg flexion from the upright standing position, followed by a quick descent to 90° knee flexion, then an explosive concentric action to reach a maximal height with hands placed on the hips. Each participant was instructed to swing their arms at a time of their choosing once the jumping motion had been initiated. These protocols have been previously validated [63].

All participants were familiar with the jumping protocols, having regularly performed jumps as part of club assessments and having participated in several practice testing sessions. The tests were conducted in an indoor environment, on a flat, non-slip surface, at a room temperature of 18–20°C, to avoid any external surface variations that might affect the results. A commercially available jump mat (SmartJump™, FusionSport, Australia) was used to perform the test. The SmartJump system has previously been validated [61]. For each test, participants performed three attempts with a 2-minute recovery interval between each trial. The best result from each participant was recorded in centimeters (cm) for further analysis.

### Reactive Agility Test (RAT)

The Reactive Agility Test (RAT) was performed according to the protocol described previously by Trajkovic et al. [62]. In the current study, the RAT involved a decision-making element provided by a light stimulus. When the participants passed the first gate, the signal showed a right or left direction. The participants then reacted to the visual signal, change direction, to pass the third gate within a Yshaped course. Participants were instructed to recognize the cues as fast as possible and not to anticipate. The total time was recorded for each trial, and the best performance was taken for analysis. The completion time was recorded using photocell gates (Microgate, Polifemo Radio Light, Bolzano, Italy) placed 0.4 m above the ground, with an accuracy of 0.001 s. All physical performance tests (SJ, CMJ, RAT) were performed in the same sequence for all participants. The standardized order was as follows: SJ, CMJA and finally RAT.

### Sleep Assessment

#### The Sleep Diary

Daily-monitored sleep quantity was assessed with the Consensus Sleep Diary (CSD) [63] which provided the following dependent sleep variables: sleep onset latency (SOL), total sleep time (TST), wake after sleep onset (WASO), subjective perception of the quality of sleep (SSQ) (often on a scale from 1 to 5 and 5 indicating high sleep quality) and sleep efficiency (SE). The SE percent is calculated as (time in bed/total sleep time) * 100 for each night.

### Spiegel Sleep Quality Perception Questionnaire

Subjective sleep quality was also assessed using the Spiegel Sleep Quality Perception Questionnaire [64] completed in the morning following days 1, 3, and 5 of the experimental conditions. Participants answered six items, with scores that ranged from 0 to 5, for sleep time, quality of sleep, nocturnal awakenings, dreams, and morning form state. The total score of the Spiegel questionnaire determined subjective sleep quality.

### The Epworth Sleepiness Scale

The Epworth Sleepiness Scale (ESS) is a self-reported scale that evaluates if a person is apt to doze off or fall asleep in everyday situations. Scores above 10 indicate excessive daytime sleepiness [65].

### Statistical analysis

Statistical analyzes were performed using the SPSS software statistical package (SPSS Inc., Chicago, IL, version 16.0). Data are presented as mean ± standard deviation (SD). The Shapiro-Wilk test of normality revealed that the data were normally distributed; therefore, parametric tests were performed. A repeated measure three-factor analysis of variance (ANOVA) (2 conditions × 2 times of day × 3 cumulative exposure days) was conducted for the cognitive and physical performances. Sleep activity patterns and somnolence were assessed using repeated measures of two-way ANOVA (2 conditions × 3 cumulative exposure days). If significant main effects were found, a Bonferroni post-hoc analysis was performed. Effect sizes were calculated using partial eta squared (ηp^2^), and interpreted as small (≥ 0.01), medium (≥ 0.06), and large (≥ 0.14), following Cohen’s benchmarks (Lakens, 2013). For the main outcome variables, 95% confidence intervals (CIs) were also reported to determine the precision and practical significance of observed effects. The level of statistical significance was set at p < 0.05.

## RESULTS

### Cognitive Performance Tests

#### Simple Reaction Time (SRT)

**FIG. 2:**
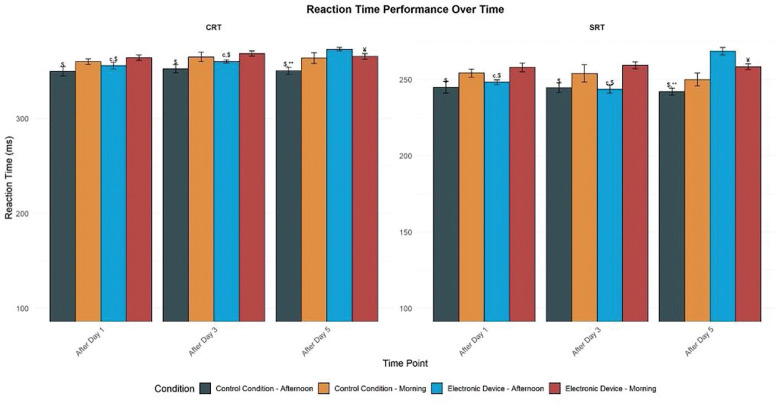
Mean (± SE) values of SRT and CRT in professional soccer players registered between 7: 00 AM and 6: 30 PM following day 1, day 3, and day 5 in electronic device and control condition SRT: Simple Reaction Time; CRT: Choice Reaction Time; M: Morning; A: Afternoon. ^c^, significant difference compared to post-day 5. $, significant difference from morning (p < 0.05). ¥, significant difference from afternoon (p < 0.05). **, significant difference between Control (C) and Experimental Group (ED) (p < 0.01).

There was a significant main effect of condition (F = 26.38, p < 0.01, d = 0.66), time (F = 3.82, p < 0.05, d = 0.25), and TOD (F = 21.71, p < 0.01, d = 0.60). Moreover, there was a significant interaction between these three factors (F = 7.20, p < 0.01, d = 0.34). In the control condition, the SRT was significantly better in the afternoon than in the morning after day 1 (p < 0.01, 95% CI [-12.12, -7.52]), day 3 (p < 0.05, 95% CI [-9.11, -1.79]), and day 5 (p < 0.05, 95% CI [−9.07, −1.34]). Similarly, with evening electronic device use, performances were better in the afternoon than in the morning only after day 1 (p < 0.01, 95% CI [-9.32, -4.94]) and day 3 (p < 0.01, 95% CI [-8.34, -4.04]). However, after day 5 in ED, the TOD effect was reversed with better performance in the morning than in the afternoon (p < 0.01, 95% CI [3.10, 9.90]). In addition, five days of night use of electronic devices reduced SRT performances significantly in the afternoon compared to post-day 1 (p < 0.01, 95% CI [-9.32, -4.94]) and to all measures of the control condition (p < 0.01, 95% CI [-12.12, -7.52]). However, this performance was similar in the morning following day 1, day 3, and day 5 and the afternoon following day 1 and day 3 in the two experimental conditions (Figure 2).

### Choice Reaction Time (CRT)

There was a significant main effect of condition (F = 12.14, p < 0.01, d = 0.44), time (F = 8.16, p < 0.01, d = 0.36), and time of day (F = 11.14, p < 0.01, d = 0.43). Moreover, these factors had a significant interaction (F = 6.11, p < 0.01, d = 0.32). In the control condition, the CRT performance was significantly lower in the afternoon than in the morning following day 1 (p < 0.01, 95% CI [-13.77, -7.79]), day 3 (p < 0.01, 95% CI [-10.52, -4.60]), and day 5 (p < 0.01, 95% CI [-8.75, -3.62]). Similarly, with evening electronic device usage, Performance was significantly higher in the afternoon than in the morning only following day 1 (p < 0.01, 95% CI [-13.57, -7.60]) and day 3 (p < 0.01, 95% CI [-11.29, -5.10]). However, following day 5, in the ED group, the time-of-day effect was reversed, with improved performance in the morning compared to the afternoon (p < 0.01, 95% CI [3.19, 9.57]). In addition, five days of night use of electronic devices reduced CRT performances significantly in the afternoon compared to post-day 1 (p < 0.01, 95% CI [-13.57, -7.60]) and to all measures of the control condition (p < 0.01, 95% CI [-13.77, -7.79]). However, this performance remained consistent in the morning following day 1, day 3, and day 5, and in the afternoon following day 1 and day 3, across both experimental conditions (Figure 2).

### Trail Making Test

#### Part A (TMT-A) (visuoperceptual abilities)

**FIG. 3:**
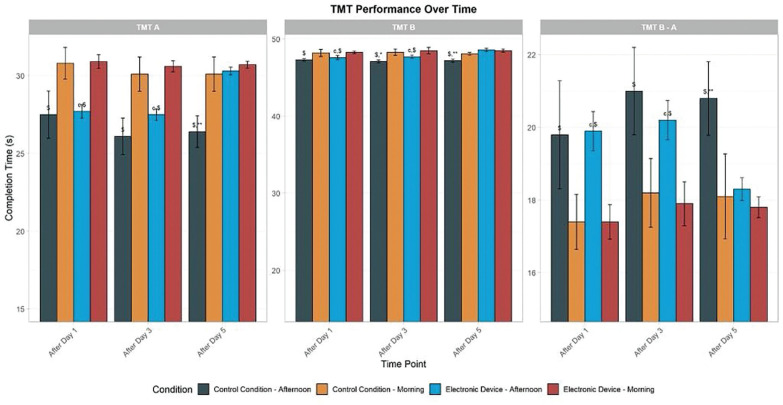
Mean (± SE) values of TMT in professional soccer players, registered between 7: 00 AM and 6: 30 PM following day 1, day 3, and day 5 in the electronic device and control condition TMT A: Trail Making Test Part A; TMT B: Trail Making Test Part B; TMT B-A: Trail Making Test (Part B – Part A); ^c^, significant difference compared to post-day 5 (p < 0.05). ^$^, significant difference from morning (p < 0.05). *Significant difference between Control (C) and Experimental Group (ED) (p < 0.05). **, significant difference between Control (C) and Experimental Group (ED) (p < 0.01).

There was a significant main effect of condition (F = 47.09, p < 0.01, d = 0.88) and TOD (F = 6.58, p < 0.01, d = 0.33) and no significant main effect of time (F = 2.32, p > 0.05, d = 0.19). Moreover, there was a significant interaction between time and condition factors (F = 7.21, p < 0.01, d = 0.34). In the control condition, the time taken to complete TMT-A was significantly shorter in the afternoon compared to the morning following day 1 (p < 0.01, 95% CI [27.2, 28.9]), day 3 (p < 0.01, 95% CI [26.4, 27.9]), and day 5 (p < 0.01, 95% CI [25.1, 26.7]). However, with evening electronic device use, performances were significantly higher in the afternoon than in the morning only following day 1 (p < 0.001, 95% CI [27.6, 28.4]) and day 3 (p < 0.01, 95% CI [27.5, 28.3]). In this condition, the TOD effect disappeared following day 5 (p > 0.05, 95% CI [-0.3, 0.1]). Furthermore, following five days of electronic device night use, the time taken to complete TMT-A decreased significantly in the afternoon compared to the control condition (p < 0.01, 95% CI [0.7, 2.3]). However, this performance was the same in the morning and the afternoon following day 1 and day 3 in both experimental conditions (Figure 3).

### Part B (TMT-B) (working memory, visual search, and taskswitching abilities)

There was a significant main effect of condition (F = 18.99, p < 0.01, d = 0.56), time (F = 3.99, p < 0.05, d = 0.25), and no significant main effect of TOD (F = 0.39, p > 0.05, d = 0.08). Moreover, there was a significant interaction between time and condition factors (F = 5.23, p < 0.01, d = 0.30). A repeated measures ANOVA revealed a significant TOD effect for TMT-B completion time under the control condition, with faster performance in the afternoon compared to the morning following day 1 (p < 0.01, 95% CI [47.4, 48.1]), day 3 (p < 0.01, 95% CI [47.3, 48.0]), and day 5 (p < 0.01, 95% CI [47.2, 47.9]). However, with evening electronic device use, performances were greater in the afternoon than in the morning only following day 1 (p < 0.01, 95% CI [47.5, 48.2]) and day 3 (p < 0.01, 95% CI [47.6, 48.3]) in this condition. The TOD effect was negligible following day 5 (p > 0.05, 95% CI [-0.5, 0.2]). In comparison with the control condition, evening electronic device use conditions reported no significant effect following day 1 and day 3 (p > 0.05, 95% CI [-0.4, 0.5]). However, TMT-B was found to be significantly lower following five days of night use of the electronic device at 5:00 PM (p < 0.01, 95% CI [0.8, 2.2]) (Figure 3).

### Trail Making Test B-A (TMT B-A)

There was a no significant main effect of condition (F = 1.75, p > 0.05, d = 0.16), time (F = 0.638, p > 0.05, d = 0.10), and no significant main effect of TOD (F = 1.50, p > 0.05, d = 0.15). Moreover, there was no significant interaction between these three factors (F = 0.32, p > 0.05, d = 0.07). In the control condition, the TMT B-A index was significantly lower in the afternoon than in the morning following day 1 (p < 0.01, 95% CI [19.6, 20.1]), day 3 (p < 0.01, 95% CI [19.2, 19.8]), and day 5 (p < 0.01, 95% CI [19.0, 19.5]). However, with evening electronic device use, the TMT B–A score was significantly lower in the afternoon than in the morning only following day 1 (p < 0.01, 95% CI [19.8, 20.3]) and day 3 (p < 0.01, 95% CI [19.5, 20.0]). The TOD effect was negligible following day 5 (p > 0.05, 95% CI [-0.2, 0.1]). In addition, following five days of electronic device night use, the TMT B-A time decreased significantly in the afternoon compared to the control condition (p < 0.01, 95% CI [0.6, 1.8]). However, this performance did not differ between the morning and afternoon following day 1 and day 3 in both experimental conditions (Figure 3).

### Attention Task (number cancellation test)

**FIG. 4:**
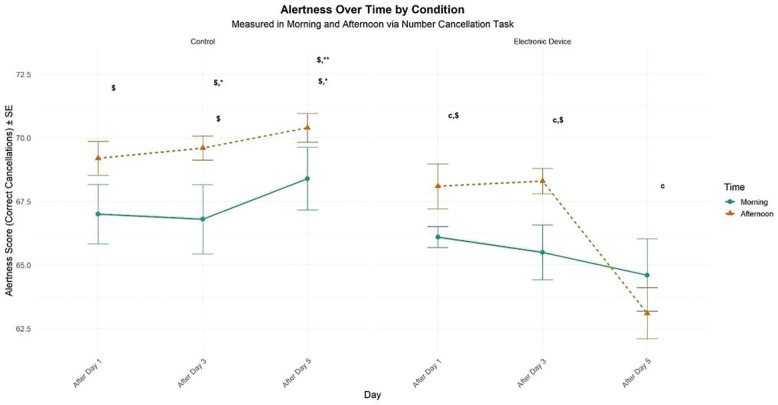
Mean (± SE) values of number cancellation in professional soccer players, registered between 7:00 AM and 6:30 PM following day 1, day 3, and day 5 in electronic device and control conditions. c, Significant difference compared to post-day 5 (p < 0.05). $, significant difference from morning (p < 0.05). *, significant difference between Control (C) and Experimental Group (ED) (p < 0.05). **, significant difference between Control (C) and Experimental Group (ED) (p < 0.01).

There was a significant main effect of condition (F = 32.76, p < 0.01, d = 0.73), TOD effect (F = 13.87, p < 0.01, d = 0.48), and no significant main effect of time (F = 2.11, p > 0.05, d = 0.18). However, there was a significant interaction between time and condition (F = 11.71, p < 0.01, d = 0.44). In the control condition, participants reported significantly higher levels of alertness in the afternoon than in the morning following day 1 (p < 0.05) and day 3 (p < 0.05). Under evening electronic device use, afternoon performance was significantly greater than morning performance only following day 1 (p < 0.01) and day 3 (p < 0.01). In this condition, the TOD effect was negligible following day 5 (p > .05). In addition, following five days of electronic device night use, the self-rating of alertness decreased significantly compared to the control condition in the morning (p < 0.05) and in the afternoon following day 3 (p < 0.05) and day 5 (p < 0.01). Furthermore, this performance did not differ between the morning following day 1 and day 3 in the two experimental conditions (Figure 4).

### Physical Performance Tests

#### Squat Jump (SJ)

**FIG. 5:**
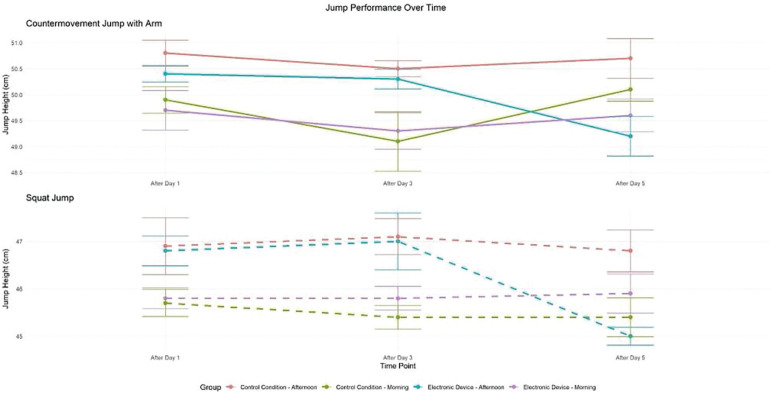
Mean (± SE) values of SJ and CMJA in professional soccer players registered between 7: 00 AM and 6: 30 PM following day 1, day 3, and day 5 in electronic device and control condition c, significant difference compared to post-day 5 (p < 0.05). $, significant difference from morning (p < 0.05). ¥, significant difference from Afternoon (p < 0.05). **, significant difference between Control (C) and Experimental Group (ED) (p < 0.01).

There was no significant effect of condition (F = 1.03, p > 0.05, d = 0.13) and a significant main effect of time (F = 3.87, p < 0.05, d = 0.25) and TOD (F = 27.04, p < 0.01, d = 0.67). Moreover, there was a significant interaction between these three factors (F = 3.48, p < 0.05, d = 0.24) and a significant interaction between time and TOD (F = 3.94, p < 0.05, d = 0.25). Under the control condition, a significant TOD effect was observed for SJ performance, with greater jump heights recorded in the afternoon compared to the morning following day 1 (p < 0.05), day 3 (p < 0.01), and 5 (p < 0.01). However, with evening electronic device use, performances were significantly higher in the afternoon than in the morning only following day 1 (p < 0.01) and day 3 (p < 0.05). In this condition, the time-of-day effect was no longer significant following day 5 (p > 0.05). In addition, after five days of night use of the electronic device, SJ decreased significantly compared to the control condition in the afternoon (p < 0.01). However, this performance did not differ between the morning and the afternoon following day 1 and day 3 in the two experimental conditions (Figure 5).

### Countermovement Jump with arm swing (CMJA)

There was a significant main effect of condition (F = 6.60, p < 0.01, d = 0.33), time (F = 4.05, p < 0.05, d = 0.25), and TOD (F = 15.39, p < 0.01, d = 0.5). Moreover, there was a significant interaction between condition and time (F = 5.96, p < 0.01, d = 0.31) and time and TOD (F = 6.79, p < 0.01, d = 0.33). In the control condition, repeated measures analysis revealed significantly greater CMJA performance in the afternoon compared to the morning following day 1 (p < 0.01), day 3 (p < 0.01), and day 5 (p < 0.05). However, with evening electronic device use, a significant TOD difference emerged, with improved afternoon performance compared to the morning only following day 1 (p < 0.05) and day 3 (p < 0.01). The TOD effect was negligible following day 5 (p > 0.05). In addition, following five days of electronic devices night use, CMJA decreased significantly compared to the control condition in the afternoon (p < 0.01). However, this performance did not differ between the morning and the afternoon following day 1 and day 3 in the two experimental conditions (Figure 5).

### Reactive Agility Test (RAT)

There was no significant effect of condition (F = 2.63, p > 0.05, d = 0.20) and a significant main effect of time (F = 3.60, p < 0.05, d = 0.25) and TOD (F = 2.77, p > 0.05, d = 0.21). Moreover, there was a significant interaction between these three factors (F = 4.93, p < 0.01, d = 0.28). In the control condition, the RA was significantly higher in the afternoon than in the morning following day 1 (p < 0.01), day 3 (p < 0.01), and day 5 (p < 0.01). However, with evening electronic device use, RA time was significantly shorter in the afternoon than in the morning only following day 1 (p < 0.01) and day 3 (p < 0.05). In this condition, the time-of-day effect was negligible following day 5 (p > 0.05). In addition, following five days of electronic device night use, RA decreased significantly compared to the control condition in the morning (p < 0.05) and afternoon (p < 0.01). However, this performance did not differ between the morning and afternoon following day 1 and day 3 in the two experimental conditions (Table 1).

**TABLE 1:** Mean (± SD) values of RA in professional soccer players, registered between 7: 00 AM and 6: 30 PM in professional soccer players following day 1, day 3, and day 5 in electronic device and control condition.

	Electronic device						Control condition					

	After day 1		After day 3		After day 5		After day 1		After day 3		After day 5	

	M	A	M	A	M	A	M	A	M	A	M	A
RA	2.4 ± 0.2	2.3 ± 0.2 ^c,$^	2.5 ± 0.2	2.3 ± 0.3 ^c,$^	2.5 ± 0.2	2.5 ± 0.2	2.4 ± 0.2	2.3 ± 0.2^$^	2.4 ± 0.2	2.3 ± 0.2^$^	2.4 ± 0.2^*^	2.2 ± 0.2^$,**^

RA: Reactive Agility Test; M: Morning; A: Afternoon.

c , significant difference compared to post-day 5 (p < 0.05).

$ , significant difference from morning (p < 0.05).

* : significant difference between Control (C) and Experimental Group (ED) (p < 0.05).

** : significant difference between Control (C) and Experimental Group (ED) (p < 0.01).

### Sleep assessment

#### Subjective sleepiness (ESS)

There was a significant main effect of condition (F = 437.58, p < 0.01, d = 3.82) and time (F = 90.13, p < 0.01, d = 1.73). Moreover, a significant interaction between time and condition was also noted (F = 114.74, p < 0.01, d = 1.95). With evening electronic device use, participants reported significantly higher subjective sleepiness on the ESS following day 3 (p < 0.01) and day 5 (p < 0.01) compared to the control condition (Table 2).

**TABLE 2:** Epworth sleepiness scale in professional soccer players across five nights; following day 1, day 3, and day 5 in electronic device and control condition.

	Electronic device			Control condition		
	Following day 1	Following day 3	Following day 5	Following day 1	Following day 3	Following day 5
ESS	6.6 ± 0.7^b,c^	11.9 ± 1.1 ^c^	13.3 ± 1.5	6.2 ± 0.8	5.9 ± 0.9^**^	6.2 ± 0.9^**^

ES: Epworth Sleepiness Scale.

b , significant difference compared to post-day 3 (p < 0.05).

c , significant difference compared to postday 5 (p < 0.05).

** , significant difference between Control (C) and Experimental Group (ED) (p < 0.01).

### Sleep diary

A main effect of the condition was revealed on TST (F = 151.85, p < 0.01, d = 2.24). Moreover, there was a significant interaction between condition and time (F = 6.32, p < 0.01, d = 0.45). The evening electronic device use significantly shortened TST. There were significant differences between groups in TST following nights 1, 3, and 5. A significant difference was also observed between groups in SOL following night 1(25.3 ± 3.9 vs 19.8 ± 4.6; p < 0.05), night 3 (29.5 ± 4.0vs 17.1 ± 5.1; p < 0.01) and night 5 (36.6 ± 6.5 vs 16.8 ± 3.0; p < 0.01) this condition. The evening electronic device use increased SOL and WASO and decreased SE compared to the control condition (see Table 3). The control group sleep quality rating was significantly higher than the evening electronic device use group following night 1 (4.3 ± 0.5 vs 3.4 ± 0.7; p < 0.05), night 3 (4.3 ± 0.6 vs 2.8 ± 0.4; p < 0.01), and night 5 (4.5 ± 0.5 vs 2.6 ± 0.5; p < 0.01) (see Table 3).

**TABLE 3:** Sleep variables in professional soccer players across five nights following day 1, day 3, and day 5 in the electronic device and control condition.

	Electronic device			Control condition		

	Following day 1	Following day 3	Following day 5	Following day 1	Following day 3	Following day 5
TST (mins)	439.4 ± 46.1 ^c^	411.8 ± 48.0	391.9 ± 48.9	497.0 ± 32.2^**^	513.7 ± 27.8^**^	518.4 ± 20.5^**^
SOL (mins)	25.3 ± 3.9 ^c^	29.5 ± 4.0 ^c^	36.6 ± 6.5	19.8 ± 4.6^*^	17.1 ± 5.1^**^	16.8 ± 3.0^**^
WASO (mins)	4.6 ± 0.5 ^b,c^	7.88 ± 1.01 ^c^	11.66 ± 1.5	2.6 ± 1.7^**^	2.4 ± 1.5^**^	2.5 ± 1.2^**^
SE (%)	88.7 ± 1.43 ^b,c^	85.74 ± 1.5 ^c^	79. 9 ± 2.3	90.9 ± 3.7^*^	91.9 ± 3.0^**^	92.4 ± 1.3^**^
SSQ (1–5)	3.4 ± 0.7 ^b,c^	2.8 ± 0.4	2.6 ± 0.5	4.3 ± 0.5^*^	4.3 ± 0.6^**^	4.5 ± 0.5^**^

TST: Total sleep time; SOL: Sleep onset latency; WASO: Wake after sleep onset; SE: Sleep efficiency; SSQ: Subjective sleep quality: the participants’ self rating of sleep quality on a 5-point Likert scale of 1 (very poor) to 5 (very good).

b , significant difference compared to post-day 3 (p < 0.05).

c , significant difference compared to post-day 5 (p < 0.05).

* : significant difference between Control (C) and Experimental Group (ED) (p < 0.05).

** , significant difference between Control (C) and Experimental Group (ED) (p < 0.01).

### Sleep Quality Assessment: Spiegel Score

The main effect of condition (F = 119.21, p < 0.01, d = 1.99) and time was revealed (F = 43.60, p < 0.01, d = 1.2). Moreover, these two factors significantly interacted (F = 47.87, p < 0.01, d = 1.263). With evening electronic device usage, the subjective sleep quality was greater on day 1 (p < 0.01) and day 3 (p < 0.01). In addition, following five days of electronic device night use, sleep quality decreased significantly compared to the control condition (p < 0.01) (see Table 4).

**TABLE 4:** Sleep Quality Assessment: Spiegel Score in professional soccer players across five nights following day 1, day 3, and day 5 in electronic device and control condition.

	Electronic device			Control condition		

	Following day 1	Following day 3	Following day 5	Following day 1	Following day 3	Following day 5
**Spiegel’s score**	**25.2 ± 1.2** ^b,c^	**23.6 ± 1.7** ^c^	**18.8 ± 1.5**	**25.4 ± 1.5**	**25.9 ± 1.4** ^**^	**25.8 ± 1.2** ^**^
Sleep latency	4.3 ± 0.7 ^c^	4.3 ± 0.6 ^c^	3.4 ± 0.6	4.1 ± 0.7	4.4 ± 0.6	4.3 ± 0.7^*^
Sleep depth	3.9 ± 0.5^b,c^	3.9 ± 0.4^c^	3 ± 0.7	4.1 ± 0.3	4.1 ± 0.3	4.1 ± 0.3^**^
Sleep duration	4.3 ± 0.6^c^	3.7 ± 0.70	3.2 ± 0.4	4.3 ± 0.4	4.2 ± 0.4^**^	4.1 ± 0.6^**^
Night-waking occurrences	4.3 ± 0.4^c^	4.1 ± 0.5	2.8 ± 0.6	4.5 ± 0.5	4.5 ± 0.6^*^	4.6 ± 0.5^**^
Night-time dreams	4.5 ± 0.5^c^	4.3 ± 0.6	3.3 ± 0.7	4.6 ± 0.5	4.7 ± 0.5	4.7 ± 0.5^*^
Morning form	3.9 ± 0.3^b,c^	3.3 ± 0.6	3.1 ± 0.5	3.9 ± 0.2	4.1 ± 0.3^**^	4.1 ± 0.3^**^

b , significant difference compared to post-day 3 (p < 0.05).

c , significant difference compared to post-day 5 (p < 0.05).

* : significant difference between Control (C) and Experimental Group (ED) (p < 0.05).

** , significant difference between Control (C) and Experimental Group (ED) (p < 0.01).

## DISCUSSION

This study examined the effects of five consecutive nights of smartphone use on cognitive and physical performance in soccer players. The main results demonstrated significant impairments with substantial effect sizes. Nocturnal smartphone use significantly reduced TST (p < 0.01, d = 2.24), decreased SE (p < 0.01), and increased SOL (p < 0.01) compared to the control condition. These sleep disruptions corresponded with significant declines in cognitive performance measures, including SRT (p < 0.01, d = 0.66), CRT (p < 0.01, d = 0.44), and TMT (TMT-A: p < 0.01, d = 0.88; TMT-B: p < 0.01, d = 0.56). Physical performance was similarly affected, with diminished SJ, CMJA (p < 0.01, d = 0.33), and RA performance. Notably, the typical TOD advantage (higher afternoon performance) observed in the control condition disappeared following five consecutive nights of smartphone exposure. While RA completion time was significantly shorter in the afternoon compared to morning sessions in the control condition across all measures (p < 0.01), this diurnal advantage was eliminated following cumulative smartphone exposure, with performance deteriorating most significantly in the afternoon following day 5 (p < 0.01). Moreover, subjective sleepiness increased progressively with smartphone use (p < 0.01), while sleep quality scores declined significantly (p < 0.01).

## Cognitive Performance

The present results indicate that prolonged exposure to blue light over several nights leads to a progressive decline in cognitive performance, particularly in tasks requiring sustained attention and quick decision-making, such as SRT and CRT tests. This decline contrasts with some earlier studies that showed immediate improvements in cognitive performance following exposure to blue light, whether at night [66, 67] or during the day [68]. While these studies report short-term benefits of exposure, the current results suggest that these initial benefits are diminished after several nights of exposure, likely due to the accumulation of cognitive fatigue induced by blue light.

Indeed, a decrease in performance throughout multiple nights of exposure, particularly in tasks involving rapid decision-making, was observed. This phenomenon aligns with the work of Scheuermaier et al. [69], who showed that the beneficial effects of blue light exposure, such as improved alertness, dissipate over time and can lead to negative cognitive performance effects after prolonged exposure. The current study results also suggest that the prolonged activation of the sympathetic nervous system due to blue light might disrupt participants’ circadian rhythms, which in turn could affect cognitive functions, as also shown by Kretschmer et al. [70] and Baek et al. [71].

Another hypothesis explaining the decline in performance after several nights of exposure could be related to the disruption of sleep. Although sleep quality was not directly measured during cognitive tasks, previous studies suggest that blue light disrupts melatonin secretion, leading to sleep disturbances and reductions in sleep quality [72]. This potential link between blue light, sleep disruption, and cognitive fatigue warrants further exploration in future studies.

The current study results do not align with the work of Tulppo et al. [73] and Alkozei et al. [74], who observed a significant reduction in reaction times following blue light exposure. This may be due to the nature of the cognitive tasks used in the present study. In contrast to SRT tests like those used in previous research of Tulppo et al. [76] and another study of Alkozei et al. [77], the present study included tasks that demand greater cognitive flexibility, such as the TMT and the D-CAT, which assess task-switching ability and cognitive flexibility [51]. Complex tasks involving sustained cognitive effort may be more likely to be negatively affected by prolonged blue light exposure due to accumulated fatigue throughout multiple nights, as suggested by Kretschmer et al. [70].

Furthermore, the present study results showed that prolonged exposure to blue light affected participants’ performance in tasks assessing visual attention and cognitive control, such as the TMT and the D-CAT, highlighting the impact on cognitive flexibility and visual attention. This progressive decline in performance could be explained by a cumulative effect of repeated exposure, which may further disrupt cognitive abilities over time, as suggested by other authors [71]. Therefore, these results underscore the importance of considering not only the immediate effects but also the long-term consequences of prolonged exposure to blue light.

## Physical performance

The current findings revealed that performance in these physical tasks was generally more effective in the afternoon than in the morning under the control condition, with no significant differences across conditions. Therefore, this suggests that physical performance may be influenced by the time of day, regardless of smartphone use.

Regarding the effects of evening smartphone exposure, the findings revealed that a single incidence of nocturnal smartphone use showed no effect on any physical performance measurements. However, repeated exposure over five consecutive nights was related to reduced jumping (SJ and CMJA) and sprint performance (RAT). This reduction is consistent with evidence demonstrating that changes in sleep quality and quantity can negatively impact physical performance. Following frequent smartphone usage, lower TST and increased WASO were connected to reductions in physical performance. Existing research supports the notion that insufficient sleep quality and duration might impair physical performance and reduced sleep duration has been demonstrated to negatively affect numerous elements of physical performance, such as aerobic capacity [75], submaximal force [76], perceived effort [77], response time [36], and execution time and accuracy [26]. Despite the limited interest that current research shows in nighttime artificial light and its direct effects on physical performance, previous studies have demonstrated that exposure to nighttime light can disrupt sleep and lead to a decrease in physical performance. For example, previous studies have found that exposure to blue-enriched light before bedtime reduces sleep duration [26] and impairs endurance performance the following day [78]. Similarly, Silvani et al. [79] reported a decrease in reaction time and muscle strength following sleep fragmentation induced by nighttime artificial light. However, Cain and Gradisar [80] suggest that the effects of light exposure may vary depending on individual chronotypes and adaptation mechanisms. The present results are consistent with those of Šmotek et al. [81], although they differ from those of Motamedzadeh et al. [82] and An et al. [67], potentially due to differences in participant characteristics or experimental conditions.

## Sleep Quality

The present findings suggest that nocturnal smartphone use significantly increased subjective sleepiness, decreased total sleep time, and impaired overall sleep quality, aligning with and extending existing literature on the effects of blue light exposure on sleep [26]. The present results are consistent with Burkhart and Phelps [83] and Souissi et al. [26], who found that blue light exposure decreased sleep quality. In contrast, some studies have reported mixed outcomes. Specifically, Viola et al. [84] observed an increase in sleep quality following blue light exposure, while other studies [85–87] found no significant change in sleep quality due to blue light exposure. Thus, while findings support the idea that blue light can detrimentally affect sleep quality, there is variability in the literature. The impact of blue light exposure on sleep quality is complex and multifaceted. While some studies suggest that blue light negatively affects sleep quality by increasing sleep latency and reducing sleep duration [26, 83, 88], other research has reported mixed findings, with some studies showing no significant changes [89, 90]. The present results support the notion that prolonged blue light exposure can disrupt sleep quality, likely due to its suppression of melatonin production. Melatonin, a hormone that regulates the sleep-wake cycle, plays a crucial role in initiating sleep and maintaining its quality. Blue light exposure, particularly in the evening, interferes with the natural circadian rhythm, delaying the onset of melatonin secretion and increasing sleep latency. This disruption in sleep can lead to reduced total sleep time and fragmented sleep, which in turn can contribute to increased fatigue and impaired cognitive performance, as observed in the current study.

The observed reduction in sleep quality following blue light exposure could be attributed to several factors, including altered circadian rhythms, decreased sleep efficiency, and increased wakefulness during the night. This disruption in sleep quality is particularly critical for athletes, as adequate sleep is essential for optimal recovery, performance, and overall health [91]. The cumulative impact of reduced sleep quality over consecutive nights of blue light exposure might exacerbate fatigue and diminish athletic performance, highlighting the importance of moderating blue light exposure before bedtime.

Regarding sleep duration, findings align with the evidence that nocturnal smartphone use reduces total sleep time. Specifically, recent studies reported a decrease in sleep duration due to blue light exposure [26, 92–94], whereas Viola et al. [84] found an increase in sleep duration. In contrast, other studies reported no significant change in sleep duration [22, 33, 85, 87, 95].

Existing evidence [96] supports the observation that blue light exposure decreases sleep duration. There are contrasting arguments considering the relevance of sleep duration for athletes. Athletes can often suffer from sleep deprivation due to training schedules [96], and blue light exposure may increase the required sleep duration. Thus, sleep deprivation may potentially worsen if blue light exposure is not moderated. However, a contrasting viewpoint may be proposed, suggesting sleep duration may not be relevant when compared to sleep quality and sleep duration may have less influence on perceived fatigue [90]. This possibly suggests that sleep duration is less relevant than sleep quality when assessing an athlete’s sleeping habits.

## Time-of-Day Effects

The shift in performance patterns observed in this study, with more pronounced declines in the afternoon compared to the morning, warrants further consideration. This time-of-day effect may partly be attributed to the interplay between circadian rhythms and blue light exposure [26]. Research has demonstrated that cognitive performance and alertness are influenced by the time of day, with afternoon performance often showing a normal decline due to circadian variations [69]. This decline suggests that blue light exposure may potentially interact with the circadian rhythm, leading to compounded effects on performance.

For instance, blue light exposure is known to affect circadian rhythms by suppressing melatonin production, thereby influencing sleep-wake cycles and alertness levels. If blue light exposure persists over consecutive nights, as in the present study, it may disrupt the individual’s circadian phase, leading to greater performance decrements during times of natural circadian low points, such as the afternoon. This interaction between blue light exposure and circadian rhythms may partly explain the decline in afternoon performance assessments, as the combined effect of circadian rhythm fluctuations and blue light disruption could further impair cognitive and physical capabilities. These interpretations align with research indicating that blue light can adversely affect sleep quality and circadian rhythms [26, 97], potentially leading to reduced performance, as observed in this study.

## Practical Applications

The findings of this study emphasise the negative impact of prolonged nighttime blue light exposure on cognitive and physical performance, as well as sleep quality in soccer players. To mitigate these effects, athletes should minimize screen exposure before bedtime by reducing smartphone use or activating blue light filters. Maintaining good sleep hygiene, including consistent sleep schedules and optimal sleep environments, is crucial for recovery and performance. Given the observed time-of-day effects, training and competition schedules should consider individual circadian rhythms, as blue light exposure may disrupt afternoon performance advantages. Long-term monitoring of sleep and performance using wearable trackers can help identify individual responses to blue light exposure. Additionally, educating athletes on the risks of nighttime screen use and promoting alternative relaxation techniques may improve sleep quality and prevent performance decrements. Coaches and sports scientists should integrate these strategies into training regimens to optimize recovery, maintain cognitive sharpness, and sustain peak physical performance throughout the season.

## Limitations and future recommendations

The present study has several methodological limitations that warrant consideration. The relatively small sample size (n = 16) limits statistical power and generalizability, particularly when considering the inter-individual variability in sleep patterns and responses to blue light exposure. The reliance on subjective sleep assessments (Epworth sleepiness scale and sleep diary) provides valuable insights but lacks the precision of objective measures like actigraphy or polysomnography. The absence of physiological marker assessment, particularly melatonin and cortisol levels, limits the ability to elucidate the underlying mechanisms of the observed effects. While the present study hypothesized that melatonin suppression plays a key role, without direct measurement, these mechanisms remain speculative. Although many variables were, factors such as screen brightness, viewing distance, and content accessed on smartphones were not standardized across participants, potentially introducing uncontrolled variability. Finally, the exclusively male sample restricts generalizability of the findings to female athletes, a limitation primarily resulting from recruitment challenges. Future research should address these limitations through several approaches. Larger-scale studies with diverse populations would enhance generalizability and enable investigation of individual differences in susceptibility to evening blue light exposure. The incorporation of objective sleep measures (polysomnography, actigraphy) alongside physiological markers would provide more comprehensive insights into both outcomes and mechanisms. Longitudinal designs extending beyond five days are essential to determine whether the observed impairments persist, worsen, or plateau over extended periods of exposure. Such research is crucial for developing evidence-based guidelines to mitigate potential adverse effects in athletic populations.

From a practical perspective, these findings suggest several implications for athletic performance management. A recommendation of the use of blue light filters or blue light-blocking glasses during evening hours to minimize circadian disruption and support better sleep hygiene would be prevalent. Additionally, coaches and sports practitioners should consider aligning training schedules with athletes’ natural sleep-wake cycles, avoiding late-night training sessions or screen-based activities close to bedtime. These strategies may help optimize recovery and performance, particularly during periods of intense training or competition. Future research should evaluate the effectiveness of these interventions specifically in athletic populations, providing empirically-supported protocols for practitioners.

## CONCLUSIONS

The present study revealed a significant negative effect of evening smartphone use on sleep quality and athletic performance in elite soccer players. The cumulative impact of consecutive nights of pre-bedtime smartphone use significantly affected both cognitive and physical performance, with noticeable effects during afternoon sessions. While completely avoiding smartphones in modern sports is unrealistic, the present findings suggest the need for thoughtful management of device use, especially during critical training and competition periods. The present study emphasizes the need for future research to explore whether similar effects are observed across diverse populations and contexts. In particular, caution is warranted in generalizing these findings beyond elite male soccer players, as the effects may differ in athletes of other sports, female athletes, or younger and older age groups. The pronounced time-of-day variations in performance indicate that training schedules may benefit from adjustments when athletes have experienced evening screen exposure, with technical and high-intensity sessions potentially being more effective during morning hours. Beyond basic performance metrics, the current results highlight the importance of finding a practical solution between modern athletic demands and optimal performance preparation, suggesting that strategic timing of device use and training sessions could help maintain peak athletic performance. The future of athletic performance optimization lies in developing strategies that recognize both the necessity of digital connectivity in modern sports and the fundamental importance of quality sleep and proper recovery.
